# Dislocation of the Femur into the Ischiatic Notch

**Published:** 1855-09

**Authors:** Frank H. Hamilton

**Affiliations:** Professor of Principles and Practice of Surgery in the Medical Department of the University of Buffalo


ART. II.—*Dislocation of the Femur into the Ischiatic Notch. Reduction by Manipulation.* By FRANK H. HAMILTON, M. D., Professor of Principles and Practice of Surgery in the Medical Department of the University of Buffalo.

In my report on "Dislocations" made to the New York State Medical Society in February last, and just published, I have stated that in reference to the reduction of dislocations of the hip by "manipulation" alone, I did not feel authorized to speak authoritatively, having as yet had no experience in this mode. I ventured, however, to express a hope, based upon the testimony before me, that it might hereafter prove, in a majority of cases, both safe and practicable. Since then an opportunity has been presented which has enabled me, in some measure, to determine, by personal experience, the value of this procedure, and I hasten to lay the case before the profession.

*March* 23, 1855. Charles McCormick, aged 21 years, at work for the "State Line R. R. Co.," was caught between two freight cars, with his back resting against one and his right knee against the other; his thigh being raised to a right angle with his body. As the cars came together he felt a "cracking" at his hip joint, and was immediately unable to walk or stand.

Two hours after, I saw McCormick, and assisted by my son Theodore, and Austin Flint, Jr., I examined the limb and made arrangements for its reduction. The patient was lying upon his back and left side. His right thigh was flexed upon h s body to nearly a right angle and adducted, the knee being carried across the opposite thigh. It was also rotated inward, but not forcibly.

Turning the lad upon his back, and raising the left leg to a position corresponding to the right, both legs were carefully measured with a tape line from the anterior superior spinous process to the patella, and the right leg was found to be shortened one and a half inches. Measuring again from the ant. sup. spin. p. to the most prominent point of the trocanter major, the distance on the dislocated limb was six inches, and on the sound limb five inches. The head of the bone could not be felt, but no doubt remained as to its position. The limb was nearly immovable, except in one direction. It could neither be abducted, or rotated outward or carried downward.

*Procedure.* The patient lying upon his back, I seized the right leg and thigh with my hands, the leg being moderately flexed upon the thigh, and carried the knee slowly up towards the belly until it had approached within twelve or fifteen inches, when, noticing a slight resistance to farther progress in this direction, I carried the knee across the body outward until I again encountered a slight resistance, and immediately I began to allow the limb to descend. At this moment a sudden slip or snap occurred near the joint, and I supposed reduction was accomplished; but on bringing the limb down completely I found it was in the same position as before. I think the head had slipped off from the lower lip of the acetabulum, after having been gradually lifted upon it.

Without waiting, I commenced to repeat the manipulation, and in precisely the same manner. Again at the same point, when the limb was just beginning to descend, a much more distinct sensation of slipping was felt, and on dropping the limb it was found to be in place and in form, with all its mobility completely restored.

No anæsthetic was employed, and no person supported the body or interfered in any way to assist in the reduction. No outcry was made by the patient, yet he informs me that moving of the limb hurt him considerably. The amount of force employed by myself was just sufficient to lift the limb, and the time occupied in the whole procedure was only a few seconds.

After the reduction, he remained upon his back, in bed, eleven days, in pursuance of my instructions. At the end of this time he began to walk about, but was unable to resume work until after eight weeks or more. It is probable that he could have walked immediately after the reduction, without much if any inconvenience, so slight was the inflammation which resulted from the accident. He never complained of pain, but upon interrogation he replied that there was a slight soreness back of the trocanter, near the head of the bone. This soreness continued several weeks and was especially present when he bent forward. Even at the present time, four months after the accident, he occasionally feels a pain at this point when he is stooping. The motions of the joint are, however, free, and he walks nimble and without any halt.

In short, if I may judge correctly from a single example, nothing could be more complete than the triumph of this process over a dislocation hitherto so formidable. Nothing could be more simple and easy of execution, and nothing more gratifying both to the surgeon and to his patient. Unless, therefore, experience shall demonstrate in its practical working defects or dangers which I cannot now anticipate, I shall regard it hereafter as one of the most valuable contributions to our art, and its inventor as a true public benefactor.

